# Transitional Care Units in the United States: A Model for Improving Dialysis Care

**DOI:** 10.34067/KID.0000000899

**Published:** 2025-06-16

**Authors:** Louis G. Baeseman, Samantha Gunning, Bharathi V. Reddy, Rita L. McGill, Arlene B. Chapman

**Affiliations:** Section of Nephrology, Department of Medicine, University of Chicago, Chicago, Illinois

**Keywords:** chronic dialysis, chronic hemodialysis, dialysis, hemodialysis, peritoneal dialysis

## Abstract

ESKD incidence rates are increasing, and mortality rates remain high. Fewer than 15% of patients use home dialysis modalities or receive preemptive kidney transplants. To address these shortcomings, executive order 13,879 (the Advancing American Kidney Health Initiative) directs the Centers for Medicare & Medicaid Services to encourage home dialysis and increase access to kidney transplants. Transitional care units (TCUs) have the potential to promote these goals by filling the gaps in care. TCUs are outpatient dialysis units for patients initiating dialysis with little or no predialysis care that provides education, facilitates smooth transitions to home or in-center dialysis, and expedites referrals to transplant clinics. TCUs offer patient-centered education, enhanced case management, and emotional support. This can improve vascular access outcomes, home dialysis utilization, and transplant referral rates. TCUs potentially address barriers to home dialysis and ideally compensate for inadequate pre-ESKD care. We performed a narrative review of several studies concerning the effect of TCUs on home dialysis utilization and patient outcomes. Eight primary studies from the United States, Canada, and the United Kingdom were reviewed, with about 7400 patients from several health and payer systems. We focused on TCU programs representing multiple payer systems, variable cohort selection criteria, and manuscripts that addressed Centers for Medicare & Medicaid Services quality measures and/or patient-centered outcomes. We call attention to the small numbers of TCUs in the United States and suggest that expansion of TCU's could benefit patients, particularly those who start dialysis under urgent conditions, which could promote equity within the ESKD population.

## Introduction

**Figure 1:**
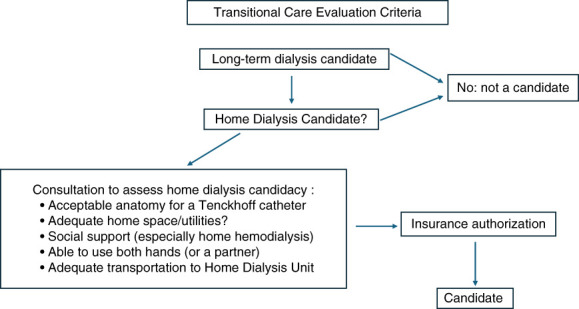
**Flow diagram for transitional care enrollment process.** Selection bias is present in the UCMC TCU admission process due to requirements for home dialysis candidacy and insurance coverage. The requirements listed above follow closely with recommendations for home dialysis candidacy per KDIGO. KDIGO, Kidney Disease Improving Global Outcomes; TCU, transitional care unit; UCMC, University of Chicago.

Transitional care units (TCUs) revive an early innovation to improve dialysis care. ESKD affects 815,000 Americans, including 470,000 patients who receive hemodialysis in facilities outside of the home.^1^ Among 65,000 patients who treat at home, >97% use peritoneal dialysis (PD), which is less complicated and expensive than home hemodialysis (HHD).^1^ Patients receiving home dialysis have 9% 1-year mortality compared with 18% among those receiving in-center dialysis (Figure 1).^1,2^

ESKD usually arises from longstanding CKD. Ideally, patients with CKD have a nephrologist and are introduced to kidney treatment options before ESKD occurs. Only 30% of patients with ESKD meet the US Centers for Medicare & Medicaid Services (CMS) optimal start criteria, defined as “preemptive kidney transplant, home-based dialysis modality, or in-center dialysis start with permanent vascular access.”^1,3,4^ Only 21% of patients enrolled in both Medicaid and Medicare achieve optimal starts.^1^ Pre-ESKD education and nephrology care are critical to having a CMS optimal start. Unfortunately, gaps in the care for patients with CKD result in incomplete patient education and low uptake of home dialysis modalities and a distressingly high rate of patients who “crash” into ESKD without appropriate preparation. Crash start dialysis, informally defined as less than 6 months of nephrology care or no exposure to a nephrologist before ESKD, is associated with inferior outcomes.^5^ This is surprisingly common; 30% of patients start dialysis under urgent conditions, defined as one or fewer visits with a nephrologist before dialysis start.^5^ Gaps in care during the complicated transition from CKD to ESKD contribute to the high early mortality rates in ESKD.^1,5^ Metcalfe *et al.* demonstrated a 90-day mortality of 12% in patients starting dialysis under urgent conditions, compared with 3% among patients who initiated with a dialysis plan (*P* < 0.05).^6^ Moreover, unplanned dialysis starts pose barriers to optimal practices, such as vascular access and referral to transplant.^7^

Unplanned starts pose a particular obstacle to home dialysis because of a lack of preparation for dialysis access and limited modality education. When dialysis starts within the time-limited inpatient setting, home dialysis is less feasible. Hospital infrastructure focuses on length of stay and is designed to efficiently funnel patients to outpatient care plans, which is most easily achieved by in-center hemodialysis. Compared with patients with planned dialysis starts, those who enter the system with a crash start are less likely to receive home dialysis 1 year later (odds ratio, 0.16; 95% confidence interval, 0.15 to 0.16),^1^ and have a reduced chance of being referred for transplant evaluation and having early transition to optimal vascular access.^1,5^

TCUs are designed with the goal of addressing gaps in care, focusing on the transition from CKD to ESKD. TCU programs can provide a tailored, patient-centered educational program that can be delivered within a dialysis facility, sometimes using a specialized area with dedicated staff. Many of the largest TCU programs are presented in Table 1. This narrative review discusses eight reported programs, providing a broad view of how TCUs may function across payer systems for a variety of patients, with acknowledgment that selection biases in TCU enrollment may influence the reported outcomes.

**Table 1:** Baseline characteristics and selected outcomes of transitional care unit programs

Characteristics	Transitional Care Program							
	UCMC	RSP	FMC	ShareHD	OR	HRH	IMPACT	Toronto
Cohort population, *N*	16	4308	724	586	93	180	1212	228
Cohort age, yr	49	62	—	63	44	66	64	55
Male sex, %	31	56.1	60.9	62	—	—	58.2	49
ESKD etiology diabetes, %	37	58.7	51.7	40	—	—	47.2	17
Black, %	82	34.4	43	14.4	—	—	36.1	—
Latino, %	0	—	16	—	—	—	20.4	—
White, %	18	59	49.3	84.6	—	—	43.5	—
AV fistula/Graft at start, %	0	28	22.4	—	—	—	18	—
Venous catheter at start, %	100	72	77.6	—	—	—	82	—
Transition to permanent access, %	67	32.6	70	—	—	—	65	—
Albumin <3.0 mg/dl, %	19	—	—	—	—	—	0	—
Home dialysis, %	37.5	—	28.4	—	30	39	—	31
Transplant waitlist, %	75	—	56.7	—	4	—	—	—

AV, arteriovenous; FMC, Fresenius Medical Care; HRH, Humber River Hospital; IMPACT, Incident Management of Patients, Actions Centered on Treatment; OR, Orientation Unit; RSP, “RightStart” Program; Toronto, Education after Acute-Start Dialysis; UCMC, University of Chicago; —, data not available.

## TCUs

The first TCU was started by Dr. Joel Eschbach in 1981, after noting that home dialysis utilization had decreased to <50% of ESKD patients at the Northwest Kidney Center.^8^ The Dialysis Orientation Unit was developed to support new patients with ESKD and increase home dialysis utilization. The focus of this TCU was intense education and “rehabilitation” over a 2-month period. The rehabilitation program focused on adapting the patient's lifestyle (dietary habits, fluid restriction) to hemodialysis, including introducing dialysis as a part of daily life that could be incorporated into the home setting. In the first year of the program, 62% of participants transitioned to home dialysis. After 10 years, home dialysis rates declined to 25%.^1^ This project included support with paid home assistants to facilitate HHD, a resource which most home dialysis programs are unable to offer.

The United Kingdom Kidney Association developed a national quality improvement program called the “ShareHD” program in 1988, intended to increase independent performance of dialysis procedures by patients who had already initiated hemodialysis in a dialysis facility.^9^ ShareHD provided system-wide standardized educational materials. The education focused on patient self-preparation for dialysis, machine preparation, and tasks performed during and after dialysis treatments, in a Plan-Do-Study-Act framework. Patient preparation included measuring their own daily weights, BPs, and temperatures. Patients were also taught proper hand washing skills and aseptic care of their vascular access sites. Ultimately, patients were encouraged to learn self-cannulation. Machine preparation included programming the dialysis prescription and starting dialysis treatments. Patients learned to respond to machine alarms, apply pressure on needle removal sites, and administer their erythropoietin injections. The primary outcome was a composite outcome of (*1*) successfully completing five or more tasks independently, (*2*) completely independent in-center hemodialysis, or (*3*) successfully transitioning to HHD. Completely independent in-center dialysis was defined as those patients who could complete all tasks without assistance from the dialysis center team. A similar program in the United States has been approved and funded by the CMS but has been traditionally underutilized. Patients in the ShareHD program achieved the composite outcome 52.3% of the time compared with 45.6% in the control group (*P* = 0.01).

A 2014 abstract from the Canadian Society of Nephrology described a six-chair TCU and a predialysis education program at Humber River Hospital in Toronto, Canada—a program with a baseline high rate of HHD uptake.^10^ Overall, home dialysis was selected by 39% of patients; 49% of the TCU cohort selected home dialysis, compared with 15% among non-TCU patients (*P* < 0.05). Patients with crash start dialysis, who would not be expected to select home modalities often under ordinary conditions, had a 50% home dialysis uptake, comparing favorably with the 25% overall rate of home dialysis uptake in Ontario,^11^ and the abysmal 5% rate of home dialysis uptake among crash start dialysis patients in the United States.^1^

Fresenius Medical Care (FMC) piloted the “RightStart” program (RSP), in which TCU “pods” were embedded into in-center dialysis facilities (Table 1).^12,13^ The initial cohort (*N*=918) was expanded in 2005, reaching 5042 incident patients. Outcomes of TCU patients were compared with control patients matched for age, sex, and ESKD diagnosis selected from the same facility within the preceding year. Matched controls could not be selected for 15% of TCU patients, raising the possibility of selection bias.^12,13^

The TCU program facilitated access to education, care navigation, promotion of durable dialysis access, and transplantation referrals. Technicians, nurses, social workers, financial coordinators, and nephrologists supported the delivery of a 4-week program to patients. Education in the first week focused on reassurance and stabilization. The second week focused on home dialysis modalities and transplantation referral. The third week emphasized modality education and durable dialysis access. Patients graduated from the TCU after 4 weeks and were enrolled to home treatment or in-center dialysis.^12^

The RSP reported a 29% survival benefit in TCU patients compared with the overall FMC incident cohort and 22% benefit compared with the matchable cohort. The RSP also examined CMS quality metrics, including hematocrit, urea reduction ratio, albumin, and catheter use.^10,11^ All secondary quality measures in the RSP cohort demonstrated small but significant improvements at 3 months compared with controls, with attenuation of effects over the next year.

A more recent study enrolled 724 incident patients in FMC TCUs between October 2019 and September 2020, each of whom was propensity-matched to four non-TCU patients. In contrast to the previous studies, a significant survival benefit was not observed. However, TCU patients selected home dialysis more often (26% versus 10%, *P* < 0.001) with 15.7% selecting PD and 9.9% selecting HHD, compared with 7.3% selecting PD and 2.8% selecting HHD among controls. Transplantation referrals were more frequent (57% versus 42%, *P* < 0.001) in the TCU cohort, although waitlist appearance did not differ. Among TCU patients who chose in-center care, transition from catheter access occurred more than controls (70% versus 63%, *P* = 0.003).^14^

DaVita developed the Incident Management of Patients, Actions Centered on Treatment (IMPACT) program as a pilot across 44 facilities between September 2007 and December 2008 (Table 1).^15^ Mortality, anemia, albumin, dialysis adequacy, and vascular access were analyzed retrospectively. IMPACT patients (*N*=1212) were matched 2:1 with propensity-matched controls from non-IMPACT sites. The IMPACT intervention modified the intake, education, and management processes. Anemia, vascular access, dietary, and socioeconomic needs were assessed during intake, with consultations expedited by the facility staff. A master checklist of tasks and goals was reviewed administratively at prespecified time points with specific interventions based on the achievement of goals. At 90 days, hemoglobin was 12.2 g/dl, compared with 12.0 g/dl; no other early outcomes differed. Arteriovenous access was more likely in the IMPACT group than controls (0.65 versus. 0.52 at 270 days and 0.63 versus 0.48 at 360 days, *P* < 0.001 for both). Reduced mortality in the IMPACT cohort was significant at 360 days (17.8% versus. 23.0%, *P* = 0.01).

Predialysis education programs may work in combination with TCUs. The University of Toronto developed an in-hospital education program for urgent start patients. From 2005 to 2009, 228 (48%) of 473 incident patients received a multimedia package focusing on dialysis modality education (Table 1).^16^ When educated, 71 (31%) elected home dialysis (49 PD and 22 HHD). Before program implementation, 87% of urgent start patients were discharged with in-center hemodialysis. An important characteristic of this cohort was that 24% of patients were starting dialysis in the setting of allograft failure. Transplant patients are often elected for adherence and favorable clinical characteristics, so selection bias may have played a role in improved outcomes.

## University of Chicago TCU Program

**Figure 2:**
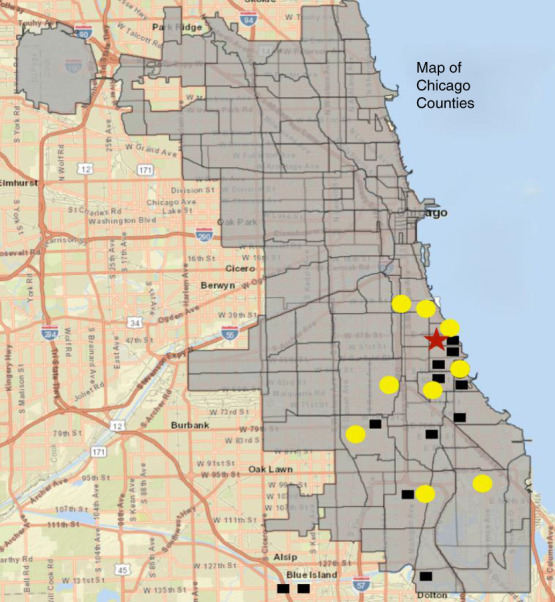
**Map of the metro Chicago area, showing overlap between patients who were enrolled in the UCMC TCU (yellow circles) and those who did not choose to enroll.** The red star marks the home dialysis facility, allowing estimation of the distance between the facility and patient addresses. Distance >15 miles appeared to pose an impediment to enrollment. The map shows that the TCU primarily enrolled patients from traditionally underserved low-income South Chicago communities.

The University of Chicago (UCMC) initiated a pilot TCU program during the coronavirus disease 2019 pandemic that focused on patients who initiated dialysis under urgent conditions. The TCU program is located in a building which houses both in-center and home dialysis programs (Figure 1). The 6–8-week pilot program was designed to facilitate education and home dialysis uptake. The data presented here are aggregate, and our Institutional Review Board reviewed with approval for publication with waived consent. All patients initiated care with thrice-weekly intermittent hemodialysis according to DaVita protocols. The primary intervention was enhanced patient to nurse, technician, and nephrologist ratios. Education focused on ESKD knowledge and home dialysis. TCU patient recruitment was performed during the hospitalization in which dialysis was initiated. Our patient cohort was predominantly Black (82%), with a primary ESKD diagnosis of diabetes in 37%. The mean age at the time of initiation of ESKD was 49 years, and 68% were female (Table 2). Of 36 patients evaluated in the inpatient setting, 16 were enrolled in TCU. Distance to TCU was an important factor in acceptance (Figure 2). The primary reason for nonenrollment was a payer incompatibility that has subsequently been resolved. Six of 16 patients (38%) subsequently transferred to the home dialysis program, and 12 of 16 (75%) successfully completed an initial transplant evaluation (Table 2). This is particularly encouraging compared with RSP outcomes, in which increases in transplant evaluation did not translate into increased waitlisting. Patients who elected home dialysis lived closer to the TCU than patients who elected in-center care (4.8 versus 12.0 miles; 95% confidence interval, 1.03 to 13.5; *P* = 0.002), implying that access to transportation was a significant barrier. Among the six home dialysis enrollees, three chose PD, one patient transitioned to in-center hemodialysis, one patient died, and one patient obtained a kidney transplant. Restricting our enrollment to patients who would consider home dialysis incorporated a selection bias for home dialysis uptake, as well as all of the favorable characteristics of self-care patients. Therefore, our data do not provide evidence that TCU care would be advantageous to patients who would not consider home dialysis.

**Table 2:** University of Chicago Transitional Care Pilot Program Data

Characteristic	TCU (*n*=16)	Non-TCU (*n*=20)
Age, yr, mean (SD)	46 (12)	53 (15)
Black, %	81	90
Male sex, %	31	55
Weight, kg, mean (SD)	87.8 (29)	78.4 (20)
Dual insurance coverage, %	63	65
Primary diagnosis diabetes, %	37.5	68.8
Albumin, mg/dl, mean (SD)	3.5 (0.6)	3.2 (0.6)
Pre-ESKD nephrologist, %	81	95
Permanent access at 6 mo, %	75	—
Home dialysis, %	37.5	—

%, percent; TCU, transitional care unit; —, data not available.

## Discussion

Quality of life is frequently cited as the primary outcome of choosing home dialysis,^17–20^ but home dialysis patients also have better cardiovascular outcomes, bone-mineral health, lower hospitalization rates, and more employment compared with in-center patients.^21–27^

TCUs provide the time and attention needed for patients to navigate ESKD initiation and choose optimal treatments. Home dialysis is usually selected by patients with pre-ESKD nephrology care and private insurance, while urgent start patients often lack these advantages.^1,5^ Our TCU program at the UCMC focused on these patients, who often came from underserved and impoverished communities. These economic disadvantages precluded the detailed pre-dialysis preparation received by better-resourced patients, and our program was designed to restore equity and improve outcomes by providing this preparation after dialysis initiation. TCUs likely have a role with these patients to improve ESKD outcomes. We focused on young, nonfrail patients for whom home dialysis could minimize the effects of ESKD on lifestyles and livelihoods. Many dialysis patients must stop working due to the scheduling constraints and symptoms associated with in-center hemodialysis. Assisting these patients to enter home dialysis modalities gave them an opportunity to continue employment, with benefits for patients, their families, and to society in general (Figure 2).

**Figure 3:**
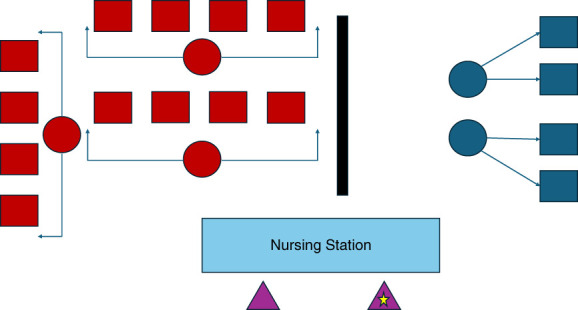
**Schematic of the UCMC TCU.** Red squares are traditional in-center dialysis patients and four represent one pod. Red circles are dialysis technicians for these pods (4:1 ratio). The dark blue squares are TCU dialysis patients in a discrete separate pod. The dark blue circles are dialysis technicians stationed near this pod. The light blue rectangle is the nursing station. The triangles are nurses that cover the unit, with one nurse focused on TCU patients reporting directly to the nephrologist.

TCU structures (Table 3) depend on staff resources in the dialysis facility to provide education and support for modality selection. Most TCUs need to have hemodialysis and PD services in a single building. Our facility structure is presented in Figure 3. We agree with other authors that an increased staff to patient ratio facilitates education, care, and closer monitoring of target weights. We favor having a designated nurse covering the TCU patients as a group, to facilitate continuity of care and a sense of community. In addition, social worker resources should be more intensively deployed in the initial period of ESKD. A dialysis program intent on developing a TCU should consider the goals of the program, whether this is home dialysis uptake, improvement of vascular access rates, facilitating transplantation, or enhancing patient experience. Targeting urgent start patients, for whom home dialysis uptake is only about 5%, seems particularly worthwhile. This is important because urgent start dialysis patients have the highest mortality of the ESKD population, even among younger patients who should have good longevity.

**Table 3:** Structure of example transitional care unit programs

Transitional Care Program								
Characteristics	UCMC	RSP	FMC	ShareHD	OR	HRH	IMPACT	Toronto
Capacity to perform HD	X	X	X	X	X	X	X	X
Capacity to provide PD/HHD training	X	—	—	—	X	X	X	X
Education staff	X	X	X	X	X	X	^a^	X
Care navigation	X	X	X	—	X	—	^a^	X
Separate area for HD	X	X	X	—	—	X	—	—
Baseline increased HD frequency	—	X	Variable	—	—	—	—	—
Time needed to achieve goals	Variable	4 wk	4 wk	12 mo	Variable	Variable	90 ds	Variable
In-hospital component	X	—	—	—	—	—	—	X
Educational materials	X	X	X	X	X	X	X	X
Limitations	Motivations/referral bias, exclusion criteria							

AV, arteriovenous; FMC, Fresenius Medical Care; HHD, home hemodialysis; HRH, Humber River Hospital; HD, hemodialysis; IMPACT, Incident Management of Patients, Actions Centered on Treatment; OR, Orientation Unit; PD, peritoneal dialysis; RSP, “RightStart” Program; TCU, transitional care unit; Toronto, In-Hospital CKD Education Program; UCMC, University of Chicago; —, did not have.

a Managers advised consultants as necessary.

An important barrier to the decision to consider home dialysis regardless of TCU availability is the availability of home dialysis clinics. Only 3344/7600 (44%) of US facilities are certified for home dialysis, of which only 635 (18%) have an active home dialysis program.^1^ Home dialysis facilities often have low censuses, suggesting underutilization, with ≤11 patients in 27% of programs. Only 7% of US programs have >50 patients.^1^ Patient decisions regarding their dialysis care could be linked to exposure to home dialysis programs,^20,28^ and opening more TCUs in cities may improve access to home dialysis care for urban patients.

Some of the observed benefits associated with home dialysis may be influenced by patient selection; offering home dialysis to a broader cohort patients may attenuate these benefits. We cannot know if the home dialysis patients selected for TCU care were simply low-risk patients who would have superior outcomes on any form of dialysis, compared with an unselected dialysis population. Encouragingly, Hingwala *et al.* in Canada found that offering PD to a broader cohort did not increase mortality or modality failure.^29^ This suggests that more patients are good candidates for home dialysis than are currently enrolled. TCUs may change entry criteria and enable more individuals to enter home dialysis without generating inferior outcomes.

All the TCUs discussed have similar limitations regarding inclusion and selection criteria. The ShareHD cohort was a national project, but patients doing well with self-care routines were those who benefited from the step wise education, while those unable to demonstrate improvements did not move forward. The RSP and other FMC programs were theoretically available for all patients admitted to FMC facilities with TCUs during the trial periods, but admission criteria could nonetheless be affected by patient and provider biases in each facility. Similarly, the UCMC program incorporates the requirement of being a willing and viable candidate for home dialysis. However, this strategy targeted patients who faced considerable social and economic disadvantages limiting their access to optimal care, who otherwise may have been lost to the in-center dialysis system. Focusing our limited resources on high-risk patients potentially ignored other groups who might benefit from TCU care. Understanding these limitations is important but does not negate the potential advantages of offering more comprehensive care to patients navigating the hazardous first year of dialysis. We anticipate that the results of our program will contribute to the knowledge base for implementation of home dialysis in disadvantaged communities and lead to more equity in the distribution of home dialysis.

## Conclusion

TCU programs are important, underutilized, and can be tailored to local needs to improve outcomes in ESKD. This review is a call to action for the expansion of TCUs in the United States to improve home dialysis uptake and early ESKD care.

## Supplementary Material
