# Supplementary material for: Transitional Care Units in the United States: A Model for Improving Dialysis Care
Source: Kidney360. 2025 Jun 16;6(8):1415–21. doi: 10.34067/KID.0000000899 (PMC12407136; doi:10.34067/KID.0000000899)
Supplement: Supplementary file 1 [file kidney360-6-1415-s001.pdf]

ASN American Society of Nephrology
JASN Journal of the American Society of Nephrology
KIDNEY360 Accessing Our World From Every Angle
CJASN Clinical Journal of the American Society of Nephrology

ASN Journal Disclosure Form

As per ASN journal policy, I have disclosed any financial relationships or commitments I have held in the past 36 months as included below. I have listed my Current Employer below to indicate there is a relationship requiring disclosure. If no relationship exists, my Current Employer is not listed.

L. Baeseman reports the following:
Employer: University of Chicago

I understand that the information above will be published within the journal article, if accepted, and that failure to comply and/or to accurately and completely report the potential financial conflicts of interest could lead to the following: 1) Prior to publication, article rejection, or 2) Post-publication, sanctions ranging from, but not limited to, issuing a correction, reporting the inaccurate information to the authors' institution, banning authors from submitting work to ASN journals for varying lengths of time, and/or retraction of the published work.

Name: Louis G. Baeseman
Manuscript ID: Manuscript ID (K360-2025-000106R1)
Manuscript Title: "Transitional Care Units in the United States: A Model for Improving Dialysis Care"
Date of Completion: April 29, 2025
Disclosure Updated Date: May 3, 2024

ASN
American Society of Nephrology
JASN
Journal of the American Society of Nephrology
KIDNEY360
Accessing Our World From Every Angle
CJASN
Clinical Journal of the American Society of Nephrology

ASN Journal Disclosure Form

As per ASN journal policy, I have disclosed any financial relationships or commitments I have held in the past 36 months as included below. I have listed my Current Employer below to indicate there is a relationship requiring disclosure. If no relationship exists, my Current Employer is not listed.

A. Chapman reports the following:
Employer: University of Chicago; Consultancy: Otsuka Pharmaceuticals; Sanofi Pharmaceuticals, Reata; Research Funding: NIDDK, Sanofi, Regulus,; Honoraria: Otsuka; Speakers Bureau: Otsuka; and Other Interests or Relationships: Special Emphasis Panel and Review Panel, NIH/NIDDK and SBIR; DOD Review Committee.

I understand that the information above will be published within the journal article, if accepted, and that failure to comply and/or to accurately and completely report the potential financial conflicts of interest could lead to the following: 1) Prior to publication, article rejection, or 2) Post-publication, sanctions ranging from, but not limited to, issuing a correction, reporting the inaccurate information to the authors' institution, banning authors from submitting work to ASN journals for varying lengths of time, and/or retraction of the published work.

Name: Arlene B. Chapman
Manuscript ID: K360-2025-000106R2
Manuscript Title: Transitional Care Units in the United States: A Model for Improving Dialysis Care
Date of Completion: June 2, 2025
Disclosure Updated Date: May 27, 2025

ASN American Society of Nephrology
JASN Journal of the American Society of Nephrology
KIDNEY360 Accessing Our World From Every Angle
CJASN Clinical Journal of the American Society of Nephrology

ASN Journal Disclosure Form

As per ASN journal policy, I have disclosed any financial relationships or commitments I have held in the past 36 months as included below. I have listed my Current Employer below to indicate there is a relationship requiring disclosure. If no relationship exists, my Current Employer is not listed.

S. Gunning reports the following:
Employer: University of Chicago

I understand that the information above will be published within the journal article, if accepted, and that failure to comply and/or to accurately and completely report the potential financial conflicts of interest could lead to the following: 1) Prior to publication, article rejection, or 2) Post-publication, sanctions ranging from, but not limited to, issuing a correction, reporting the inaccurate information to the authors' institution, banning authors from submitting work to ASN journals for varying lengths of time, and/or retraction of the published work.

Name: Samantha Gunning
Manuscript ID: K360-2025-000106R1
Manuscript Title: Transitional Care Units in the United States: A Model for Improving Dialysis Care
Date of Completion: April 30, 2025
Disclosure Updated Date: April 30, 2025

ASN
American Society of Nephrology
JASN
Journal of the American Society of Nephrology
KIDNEY360
Accessing Our World From Every Angle
CJASN
Clinical Journal of the American Society of Nephrology

ASN Journal Disclosure Form

As per ASN journal policy, I have disclosed any financial relationships or commitments I have held in the past 36 months as included below. I have listed my Current Employer below to indicate there is a relationship requiring disclosure. If no relationship exists, my Current Employer is not listed.

R. McGill reports the following:
Employer: University of Chicago Medicine; Consultancy: AVA Health; Ownership Interest: CVS Health (spouse's former employer);; and Advisory or Leadership Role: Editorial Board Member, Journal of Vascular Access (unpaid); UNOS LIving Donor Committee (unpaid).

I understand that the information above will be published within the journal article, if accepted, and that failure to comply and/or to accurately and completely report the potential financial conflicts of interest could lead to the following: 1) Prior to publication, article rejection, or 2) Post-publication, sanctions ranging from, but not limited to, issuing a correction, reporting the inaccurate information to the authors' institution, banning authors from submitting work to ASN journals for varying lengths of time, and/or retraction of the published work.

Name: Rita L. McGill
Manuscript ID: K360-2025-000106R2
Manuscript Title: Transitional Care Units in the United States: A Model for Improving Dialysis Care
Date of Completion: June 2, 2025
Disclosure Updated Date: May 8, 2025

ASN American Society of Nephrology JASN Journal of the American Society of Nephrology KIDNEY360 Accessing Our World From Every Angle CJASN Clinical Journal of the American Society of Nephrology

ASN Journal Disclosure Form

As per ASN journal policy, I have disclosed any financial relationships or commitments I have held in the past 36 months as included below. I have listed my Current Employer below to indicate there is a relationship requiring disclosure. If no relationship exists, my Current Employer is not listed.

B. Reddy reports the following:
Employer: University of Chicago; and Other Interests or Relationships: Member, Medical Review Board, ESRD Network 10 (elected).

I understand that the information above will be published within the journal article, if accepted, and that failure to comply and/or to accurately and completely report the potential financial conflicts of interest could lead to the following: 1) Prior to publication, article rejection, or 2) Post-publication, sanctions ranging from, but not limited to, issuing a correction, reporting the inaccurate information to the authors' institution, banning authors from submitting work to ASN journals for varying lengths of time, and/or retraction of the published work.

Name: Bharathi V. Reddy
Manuscript ID: K360-2025-000106R1
Manuscript Title: Transitional Care Units in the United States: A Model for Improving Dialysis Care.
Date of Completion: April 30, 2025
Disclosure Updated Date: April 30, 2025
